# Parental Knowledge and Awareness Regarding Early Orthodontic Consultation in Children: A Cross-Sectional Study

**DOI:** 10.7759/cureus.73842

**Published:** 2024-11-17

**Authors:** Mahdi Mohammed Alwusaybie, Fatima Sameer AlBaqshi, Mohammed Afif Alshaks, Mousa Zaki Alabdullah, Mohammed Abdulaziz Alwosaibei, Hassan Jumah AlBahrani, Hassan Mohammed Alsaleh, Naji Mohammad Almadeh, Ali Hussain Alyousef

**Affiliations:** 1 Department of Endodontics, Dental Centre, Hail Cluster, Ministry of Health, Hail, SAU; 2 Department of Orthodontics and Dentofacial Orthopedics, King Faisal General Hospital, Ministry of Health, Hofuf, SAU; 3 College of Dentistry, King Faisal University, Hofuf, SAU; 4 College of Medicine and Medical Science, Arabian Gulf University, Manama, BHR

**Keywords:** awareness, children, early treatment, knowledge, orthodontics, parents

## Abstract

Objective: The present study aimed to assess parental knowledge and awareness of early orthodontic consultation in children.

Methods: This cross-sectional study was conducted among parents of schoolchildren who were randomly selected from public schools in the Al Ahsa region of Saudi Arabia, using a convenience sampling technique. Data were collected using a prevalidated, pretested study questionnaire distributed to parents through the schoolchildren. The questionnaire focused on the demographic data, knowledge, and awareness of parents regarding their children’s need for orthodontic consultation. Descriptive statistics and the Chi-square test were used to analyze the quantitative data collected.

Results: More than 50% of the study participants exhibited a good perception of most of the aspects of early orthodontic consultation in children. However, only 42.8% of study subjects exhibited awareness of space maintainers and their use in a growing child, while only 37.5% knew that the first orthodontic visit should be at seven years of age. The age of the participants as well as their educational level were found to be important demographic factors influencing knowledge and awareness of the need for early orthodontic consultation (p<0.05).

Conclusion: The present study found that there were knowledge and awareness gaps among Saudi parents regarding early orthodontic consultation, including those on the need for space maintainers and the age of initial orthodontic consultation. There exists a need for a targeted focus on creating awareness amongst younger and lesser-educated parents, which can largely improve the awareness pertaining to early orthodontic treatment initiation in children.

## Introduction

The facial appearance of an individual directly relates to self-confidence, social interaction, and attractiveness, while a well-balanced esthetic occlusion is perceived as a major contributing factor to the facial appearance [1]. Individuals affected with malocclusion are prone to peer bullying and social rejection, which subsequently result in psychosocial problems [2]. Early diagnosis and management of malocclusion could benefit children as it reduces the psychological burden of facial disfigurement in the later years, shortens the treatment time, minimizes oral functional problems, and improves oral health and quality of life [3]. Malocclusion is the third most common dental condition, following caries and periodontal disease, and has been considered to be a public dental health problem with high prevalence and treatment needs [4]. This can be achieved by initiating early intervention at a specific age in the patient's life, at which point the treatment plan can be modified to control growth, thus preventing the need for complex non-surgical or surgical treatments in the future. Previous studies conducted in Saudi Arabia found that the most common molar relation was Class I with a prevalence rate of approximately 80%, followed by Class II and Class III [5-7]. These high prevalence rates leading to increased unmet treatment needs might reflect parents' lack of knowledge and awareness regarding early orthodontic consultation or care in children.

Many distinct motives sway the decision of a parent for his/her child before starting orthodontic procedures. The reasons for obtaining orthodontic treatment can be divided into esthetic, functional, or social motives [8]. Previous studies in Saudi Arabia reported that most orthodontic patients desired orthodontic treatment to improve their facial appearance [9]. The knowledge of the patient’s motivation to seek orthodontic treatment assists in developing an appropriate individualized treatment plan for each patient [10]. Furthermore, it guides the orthodontist in providing an effective patient interaction, thereby enhancing their compliance with orthodontic treatment.

Lack of knowledge or awareness among parents about the right time of initiation of early orthodontic treatment and potential consequences of malocclusion, such as the development of periodontitis, impaired oral functions involving speech and mastication, and increased risk of trauma [11], could have an impact on the oral health of children. Furthermore, increased orthodontic unmet treatment needs in children could be related to a lack of knowledge and awareness regarding early orthodontic care. This study might help to measure the parents' need for further education to increase knowledge and awareness regarding early orthodontic consultation in children, which in turn could motivate them to seek early care for children. The aim of the present study is to evaluate parental knowledge and awareness about early orthodontic consultation in children from Al Ahsa, Eastern Province of Saudi Arabia.

## Materials and methods

The present cross-sectional study was conducted among the parents of schoolchildren aged between six and 12 years selected from public schools in the Al Ahsa region of Saudi Arabia during the study period from March to June 2024. A convenience sampling technique was used to select the sample size. Random dates were chosen during the study period, and the questionnaire was distributed to children attending the respective schools on those days. The child's age at the last birthday was considered as the child's age at the time of examination. The inclusion criteria for those included in the study were male and female Saudi children aged between six and 12 years (according to school records). Exclusion criteria included children with systemic diseases and/or craniofacial anomalies, a previous history of orthodontic treatment, children whose parents were related to the dental field, and those questionnaires with incomplete data. The outcomes were the parents' knowledge of their children’s early orthodontic consultation, and the exposures were parental age, education level, and number of children.

This study used a validated and tested questionnaire adopted from a previous similar study conducted in Saudi Arabia [12]. The questionnaire was translated to Arabic officially and checked for accuracy and clarity in a pilot study on 30 subjects. This pilot study showed that the questionnaire was easy to understand and no difficulties were faced by the parents to mention their responses in the questionnaire. The questionnaire consisted of 14 items divided into two parts, with the first part focusing on the demographic data of participants while the second part assessed the knowledge and awareness of parents regarding their children’s need for orthodontic consultation and treatment. The children were asked to take the questionnaire to their parents to answer the survey and bring it back the next day. Parents desiring to respond to the questionnaire in electronic format were provided with a quick response (QR) code in the questionnaire for access to submit their responses. Prior permission was obtained from the concerned school authorities for the same. Also, written informed consent was sent along with the questionnaire to be signed by the parents for participation in the survey. Participation in the study was entirely voluntary. The protocol of this research was reviewed and approved by the Institutional Ethical Research Committee of King Faisal University, Hofuf, Saudi Arabia (Ref: KFU-2024-ETHICS2817).

The data were statistically analyzed using IBM SPSS software version 25.0 (IBM Corp., Armonk, NY, USA). Descriptive statistics and frequencies were ascertained. The Chi-square test was used to detect any statistical differences between the study variables, and a p-value of 0.05 or less was considered statistically significant.

## Results

A total of 400 questionnaires were sent through children to the parents, and all the parents replied to the questionnaires (100% response rate). Hence the final sample for analysis included 400 parental responses, of which 50.5% (n = 202) were mothers and 49.5% (n = 198) were fathers. The overall mean age of the study sample was 36.8±5.50 years (mean±SD), while the mean age of males and females was 38.3±5.24 and 35.5±5.39 years, respectively. The distribution of frequency and percentage of demographic characteristics of study participants is shown in Table 1. Among the participants, parents aged 40 years and above were higher (55.3%), followed by those aged between 30-39 years (30.5%). The majority of the participants were married (92.5%), and 57.8% of the participants were employed, while most parents had completed a bachelor’s degree. When the education level of participants was compared with the responses, a statistically significant difference was observed (p<0.05). Parents holding a bachelor’s degree showed a higher proportion of correct responses compared to those with other levels of education. However, in terms of knowledge related to the age of orthodontic treatment initiation (Q5), early primary tooth loss (Q3), and causes of malocclusion (Q2), no significant differences were noted between the groups. Only 25% of them had an education level of high school or below. Most participants reported (73%) having more than two children.

**Table 1:** Distribution of parental responses according to frequency and percentage (English version of the Arabic questionnaire)

Survey responses	Response	n (%)
Q1: Do you think a beautiful smile is important for the healthy development of a child’s personality?	No	14 (3.5)
	Yes	386 (96.5)
Q2: What are the causes of malocclusion in children?	Bad oral habits (Eg: thumb sucking etc)	55 (13.8)
	Heredity	57 (14.3)
	Both	219 (54.8)
	I don't know	69 (17.3)
Q3: If a primary molar was lost prematurely/early due to decay, what should be done?	Check the need for a space maintainer	171 (42.8)
	I don't know	57 (14.3)
	Nothing. The permanent tooth will replace it	172 (43.0)
Q4: Who will you first go to consult regarding orthodontic treatment for your child?	A general dentist	103 (25.8)
	A general physician/pediatrician	34 (8.5)
	An orthodontist	252 (63.0)
	I don't know	11 (2.8)
Q5: At what age should your child go to his/her first orthodontic consultation?	18+ years	78 (19.5)
	12-16 years	110 (27.5)
	7-11 years	150 (37.5)
	I don't know	62 (15.5)
Q6: Do you think the age of the person when starting orthodontic treatment can affect the outcome?	I don't know	33 (8.3)
	No	29 (7.3)
	Yes	338 (84.5)
Q7: Do you think children can have orthodontic treatment during their growth period?	I don't know	60 (15.0)
	No	96 (24.0)
	Yes	244 (61.0)

Table 2 shows the distribution of frequency and percentage of responses by study participants. About 70.8% of parents had responded that there was no history of previous or current experience of any form of orthodontic treatment. Most parents (96.5%) agreed that a beautiful smile could positively affect the personality of a child. While 54.8% of study subjects opined both oral habits and heredity to be the root cause of malocclusions in children, only 42.8% of participants had knowledge on the need for space maintainers in children with early primary molar loss. Although most parents (63%) suggested that they would consult an orthodontist first to identify the need for correction of malocclusion in their child, only 37.5% of the study participants knew that the first orthodontic consultation should be done when the child is between seven and 11 years of age. However, 84.5% and 61% of participants knew that the age of orthodontic treatment initiation could influence treatment outcomes and that orthodontic treatment is feasible during the growing period, respectively.

**Table 2:** Demographic details of the study participants as per frequency and percentage

Variables		Frequency	Percentage
Gender	Female	202	50.5
	Male	198	49.5
Age (in years)	18-29	57	14.3
	30-39	122	30.5
	40 or more	221	55.3
Marital status	Married	370	92.5
	Divorced	16	4.0
	Widowed	14	3.5
Employment status	Employed	231	57.8
	Unemployed	169	42.3
Educational level	High school or less	100	25.0
	Bachelor	225	56.3
	Diploma	48	12.0
	Postgraduate degree	27	6.8
No. of children	≤ 2 children	108	27.0
	> 2 children	292	73.0
History of previous or current orthodontic treatment	No	283	70.8
	Yes	117	29.3

A comparison of parental age with responses is shown in Table 3. Parents with advanced age were observed to be an important influencer of correct responses, the comparison being statistically significant between the age groups in terms of their responses for all questions related to awareness except for those on early primary molar loss and the importance of smile in a child’s personality development (p<0.05). It was disturbing to see that 57.2% of the parents had a lack of knowledge or awareness about the importance of space maintainers in managing early tooth loss in children, and the difference was not significant between the three age groups (p = 0.387). 

**Table 3:** Parental awareness regarding early orthodontic consultation based on their age (in years) Chi-square test was used; *p ≤ 0.05 indicates statistical significance

Survey question	Response	Age (in years)			Total	p-value (Chi square) (χ2)
		18-29 n (%)	30-39 n (%)	40 or more n (%)		
Q1: Do you think a beautiful smile is important for the healthy development of a child’s personality?	No	1 (0.2)	6 (1.5)	7 (1.8)	14 (3.5)	0.518 (χ2=1.31)
	Yes	56 (14)	116 (29)	214 (53.5)	386 (96.5)	
Q2: What are the causes of malocclusion in children?	Bad oral habits	5 (1.2)	18 (4.5)	32 (8)	55 (13.8)	0.001* (χ2=23.85)
	Heredity	19 (4.8)	11 (2.8)	27 (6.8)	57 (14.2)	
	Both	26 (6.5)	76 (19)	117 (29.2)	219 (54.8)	
	I don’t know	7 (1.8)	17 (4.2)	45 (11.2)	69 (17.2)	
Q3: If a primary molar was lost prematurely/early due to decay, what should be done?	Check the need for a space maintainer	19 (4.8)	57 (14.2)	95 923.8)	171 (42.8)	0.387 (χ2=4.14)
	I don’t know	7 9 (1.8)	16 (4.0)	34 (8.5)	57 (14.2)	
	Nothing. The permanent tooth will replace it	31 (7.8)	49 (12.2)	92 (23.0)	172 (43.0)	
Q4: Who will you first go to consult regarding orthodontic treatment for your child?	A general dentist	9 (2.2)	24 (6.0)	70 (17.5)	103 (25.8)	0.004* (χ2=19.38)
	A general physician/ pediatrician	10 (2.5)	5 (1.2)	19 (4.8)	34 (8.5)	
	An orthodontist	36 (9.0)	88 (22.0)	128 (32.0)	252 (63.0)	
	I don’t know	2 (0.5)	5 (1.2)	4 (1.0)	11 (2.8)	
Q5: At what age should your child go to his/her first orthodontic consultation?	18+ years	7 (1.8)	37 (9.2)	34 (8.5)	78 (19.5)	0.001* (χ2=28.96)
	12-16 years	13 (3.2)	32 (8)	65 (16.2)	110 (27.5)	
	7-11 years	34 (8.5)	39 (9.8)	77 (19.2)	150 (37.5)	
	I don’t know	3 (0.8)	14 (3.5)	45 (11.2)	62 (15.5)	
Q6: Do you think the age of the person when starting orthodontic treatment can affect the outcome?	I don’t know	4 (1.0)	4 (1.0)	25 (6.2)	33 (8.2)	0.038* (χ2=10.13)
	No	2 (0.5)	7 (1.8)	20 (5.0)	29 (7.2)	
	Yes	51 (12.8)	111 (27.8)	176 (44.0)	338 (84.5)	
Q7: Do you think children can have orthodontic treatment during their growth period?	I don’t know	5 (1.2)	15 (3.8)	40 (10.0)	60 (15.0)	0.001* (χ2=20.88)
	No	6 (1.5)	23 (5.8)	67 (16.8)	96 (24.0)	
	Yes	46 (11.5)	84 (21.0)	114 (28.5)	244 (61.0)	

When the education level of participants was compared with the responses, a statistically significant difference was observed (p<0.05). Parents holding a bachelor’s degree showed a higher proportion of correct responses compared to those with other levels of education. However, in terms of knowledge related to the age of orthodontic treatment initiation (Q5), early primary tooth loss (Q3), and causes of malocclusion (Q2), no significant differences were noted between the groups (Table 4).

**Table 4:** Parental awareness on early orthodontic consultation based on the level of education Chi-square test was used; *p ≤ 0.05 indicates statistical significance

Survey question	Response	Education level				Total	p-value
		≤ High school	Bachelor	Diploma	Postgraduate degree		
Q1: Do you think a beautiful smile is important for the healthy development of a child’s personality?	No	4 (1.0)	3(7.5)	6(1.5)	1(0.25)	14 (3.5)	0.002* (χ2=14.72)
	Yes	96 (24)	222 (55.5)	42 (10.5)	26 (6.8)	386 (96.5)	
Q2: What are the causes of malocclusion in children?	Bad oral habits	17 (4.2)	31 (7.8)	5 (1.2)	2 (0.5)	55 (13.8)	0.168 (χ2=12.88)
	Heredity	8 (2)	35 (8.8)	11 (2.8)	3 (0.8)	57 (14.2)	
	Both	52 (13)	127 (31.8)	22 (5.5)	18 (4.5)	219 (54.8)	
	I don’t know	23 (5.8)	32 (8.0)	10 (2.5)	4 (1.0)	69 (17.2)	
Q3: If a primary molar was lost prematurely/early due to decay, what should be done?	Check the need for a space maintainer	37 (9.2)	96 (24)	21 (5.2)	17 (4.2)	171 (42.8)	0.344 (χ2=6.76)
	I don’t know	18 (4.5)	30 (7.5)	6 (1.5)	3 (0.8)	57 (14.2)	
	Nothing; the permanent tooth will replace it	45 (11.2)	99 (24.8)	21 (5.2)	7 (1.8)	172 (43.0)	
Q4: Who will you first go to consult regarding orthodontic treatment for your child?	A general dentist	22 (5.5)	49 (12.3)	23 (5.6)	9 (2.2)	103 (25.8)	0.0002* (χ2=31.87)
	A general physician/pediatrician	18 (4.5)	11 (2.8)	3 (0.8)	2 (0.5)	34 (8.5)	
	An orthodontist	58 (14.5)	158 (39.5)	21 (5.2)	15 (3.8)	252 (63.0)	
	I don’t know	2 (0.5)	7 (1.7)	1 (0.3)	1 (0.3)	11 (2.8)	
Q5: At what age should your child go to his/her first orthodontic consultation?	18+ years	12 (3.0)	53 (13.2)	7 (1.8)	6 (1.5)	78 (19.5)	0.231 (χ2=11.68)
	12-16 years	32 (8.0)	57 (14.2)	15 (3.8)	6 (1.4)	110 (27.5)	
	7-11 years	36 (9.0)	88 (22.0)	16 (4.0)	10 (2.5)	150 (37.5)	
	I don’t know	20 (0.5)	27 (6.8)	10 (2.5)	5 (1.2)	62 (15.5)	
Q6: Do you think the age of the person when starting orthodontic treatment can affect the outcome?	I don’t know	12 (3.0)	12 (3.0)	6 (1.5)	3 (0.8)	33 (8.2)	0.395 (χ2=13.23)
	No	12 (3.0)	11 (2.8)	5 (1.2)	1 (0.2)	29 (7.2)	
	Yes	76 (19.0)	202 (50.5)	37 (9.2)	23 (5.8)	338 (84.5)	
Q7: Do you think children can have orthodontic treatment during their growth period?	I don’t know	23 (5.8)	26 (6.5)	6 (1.5)	5 (1.2)	60 (15.0)	0.006 (χ2=18.18)
	No	28 (7.0)	46 (11.5)	18 (4.5)	4 (1.0)	96 (24.0)	
	Yes	49 (12.2)	153 (38.2)	24 (6.0)	18(4.5)	244 (61.0)	

## Discussion

Preventive and interceptive orthodontics are proven approaches to managing developing malocclusions in the dentofacial complex. Early orthodontic consultation is of utmost importance for children as it could identify deviations in the growth of the jaws, prevent esthetic and functional concerns, and reduce the complexity of potential malocclusion. Since a child is completely dependent on his/her parents for health-related consultations, parental awareness is crucial for early orthodontic visits, and the ideal time for a child to have their first orthodontic screening visit was suggested to be around seven years by the American Association of Orthodontists [13]. Moreover, previous studies in Saudi Arabia had reported high orthodontic treatment needs among children in the mixed dentition stages [14]. This study was thus undertaken to assess the parental knowledge and awareness of the need for early orthodontic consultations in children.

The majority of the participants (96.5%) in the present study agreed that a beautiful smile plays a major role in molding a child’s personality. This was very similar to the study done by Alsaggaf et al. [12], but the proportion was higher compared to the findings reported by Aldweesh et al. [15]. Awareness of the influence of malocclusion on the psychosocial aspects of an individual’s life is an important step in ensuring early orthodontic consultations, and this proportion was very high among the parents in the present study. This finding, however, was contrary to that of Alharbi et al. [16], wherein 89.6% of the study sample disagreed with the teeth alignment playing a role in personality development. The most probable reason could be attributed to the higher education level of most of the participants in the present study.

The combination of heredity and oral habits leading to malocclusion in children has been assented by the majority of the participants in the present study, while most opined that orthodontists should be the first choice for consultations related to malalignment of teeth. Most of the parents were also well aware that the age of treatment initiation influenced outcomes in the child and that orthodontic treatment could be started during the growing period of children. Overall, most parents were observed to have reasonably good knowledge regarding early orthodontic consultation except on questions related to the use of space maintainers in premature loss of primary teeth and the age of the first orthodontic visit, which saw the percentage of correct responses on the downside among the participants. Our results were similar to those from the study by Alsaggaf et al. [12], except on questions related to primary tooth loss and the appropriate age of first orthodontic consultation, as the percentage of correct responses was low compared to the current study. These variations could be due to regional differences in the level of awareness or knowledge between population groups in Saudi Arabia. Contrary to this, Alharbi et al. [16] reported that only 4% of the study sample exhibited good knowledge of early orthodontic treatments in children.

Age and education level were observed to be two important demographic factors influencing questionnaire responses in our study. These findings were similar to those of a study by Basri et al. [17], wherein they observed an advantage of awareness in parents with increasing age and among those with postgraduate education levels. In the present study, participants with an advanced age and those with a bachelor’s degree in education gave better responses compared to other categories. Socioeconomic status was not considered in this study, as the majority of the population utilizes oral care and orthodontic services through the public-funded healthcare system in Saudi Arabia. Overall, the study demonstrates the need for a targeted focus on creating awareness regarding the aspects of early orthodontic consultation amongst the younger aged and lesser-educated parents. Organizing community-based dental health care programs with more emphasis on the importance of early dental care, including early orthodontic care in children, can largely improve the awareness pertaining to early orthodontic treatment initiation in children among the Saudi parent population.

Some limitations of the present research could include not considering the age of the child and the severity of existing malocclusion in children. These factors could have a significant influence on orthodontic treatment-related decisions taken by the parents. The study also did not include the different regions of Saudi Arabia to obtain a better perspective on the level of awareness. The present study was confined to a specific location in the Eastern province, and hence the generalizability of the findings to the Saudi population could be limited. Further studies could be undertaken in the future with better regional representation and a larger sample size.

## Conclusions

The study participants exhibited good knowledge of early orthodontic consultation of their child. However, there were knowledge and awareness gaps among Saudi parents regarding early orthodontic consultation, including those on the need for space maintainers and the age of initial orthodontic consultation in children. There exists a need for a targeted focus on creating awareness amongst younger and lesser-educated parents, which can largely improve the awareness pertaining to early orthodontic treatment initiation in children. Early orthodontic consultation and management during the mixed dentition period might reduce the need for complex treatment at later stages and could reduce the financial burden both for the parents and the healthcare provider, especially in Saudi Arabia, owing to its public health system funding for oral and orthodontic treatment.
